# Field evaluation of the 22 rapid diagnostic tests for community management of malaria with artemisinin combination therapy in Cameroon

**DOI:** 10.1186/s12936-016-1085-0

**Published:** 2016-01-20

**Authors:** Innocent M. Ali, Jude D. Bigoga, Dorothy A. Forsah, Fidelis Cho-Ngwa, Vivian Tchinda, Vicky Ama Moor, Josephine Fogako, Philomena Nyongalema, Theresa Nkoa, Albert Same-Ekobo, Joseph Mbede, Etienne Fondjo, Wilfred F. Mbacham, Rose G. F. Leke

**Affiliations:** The Biotechnology Centre, University of Yaoundé 1, BP 8094, Yaoundé, Cameroon; Department of Biochemistry, University of Dschang, Dschang, Cameroon; National Malaria Control Program, Ministry of Public Health, Yaoundé, Cameroon; Department of Biochemistry and Molecular Biology, University of Buea, Buea, Cameroon; Medical Research Centre, Institute of Medical Research and Medicinal Plant Study, Yaoundé, Cameroon; Faculty of Medicine and Biomedical Sciences, University of Yaoundé 1, Yaoundé, Cameroon; Ministry of Public Health, Yaoundé, Cameroon; Faculty of Health Sciences, Université des Montagnes, Bangante, Cameroon; Paediatrics Unit, University Teaching Hospital, Faculty of Medicine and Biomedical Sciences, University of Yaoundé 1, Yaoundé, Cameroon; Laboratory of Immunology and Parasitology, The Biotechnology Centre, University of Yaoundé 1, BP 8094, Yaoundé, Cameroon

**Keywords:** Malaria, Sensitivity, Rapid diagnostic test, Microscopy, Specificity, HRP2, Positive predictive value, *Plasmodium falciparum*

## Abstract

**Background:**

All suspected cases of malaria should receive a diagnostic test prior to treatment with artemisinin-based combinations based on the new WHO malaria treatment guidelines. This study compared the accuracy and some operational characteristics of 22 different immunochromatographic antigen capture point-of- malaria tests (RDTs) in Cameroon to inform test procurement prior to deployment of artemisinin-based combinations for malaria treatment.

**Methods:**

One hundred human blood samples (50 positive and 50 negative) collected from consenting febrile patients in two health centres at Yaoundé were used for evaluation of the 22 RDTs categorized as “Pf Only” (9) or “Pf + PAN” (13) based on parasite antigen captured [histidine rich protein II (HRP2) or lactate dehydrogenase (pLDH) or aldolase]. RDTs were coded to blind technicians performing the tests. The sensitivity, specificity, and predictive values of the positive and negative tests (PPV and NPV) as well as the likelihood ratios were assessed. The reliability and some operational characteristics were determined as the mean values from two assessors, and the Cohen’s kappa statistic was then used to compare agreement. Light microscopy was the referent.

**Results:**

Of all RDTs tested, 94.2 % (21/22) had sensitivity values greater than 90 % among which 14 (63.6 %) were ‘Pf + PAN’ RDTs. The specificity was generally lower than the sensitivity for all RDTs and poorer for “Pf Only” RDTs. The predictive values and likelihood ratios were better for non-HRP2 analytes for “Pf + PAN” RDTs. The Kappa value for most of the tests obtained was around 67 % (95 % CI 50–69 %), corresponding to a moderate agreement.

**Conclusion:**

Overall, 94.2 % (21/22) of RDTs tested had accuracy within the range recommended by the WHO, while one performed poorly, below acceptable levels. Seven “Pf + PAN” and 3 “Pf Only” RDTs were selected for further assessment based on performance characteristics. Harmonizing RDT test presentation and procedures would prevent mistakes of test performance and interpretation.

**Electronic supplementary material:**

The online version of this article (doi:10.1186/s12936-016-1085-0) contains supplementary material, which is available to authorized users.

## Background

The challenge of controlling malaria is a continuous reality in most sub-Saharan countries. Despite increasing efforts in prevention and treatment, malaria has remained a major cause of morbidity and mortality, with an estimated 451 million clinical cases of malaria in 2007 alone, mostly in sub-Saharan Africa [1]. In Cameroon, malaria continues to be endemic with estimated 71 % living in high transmission areas [2], and the major cause of morbidity and mortality among the most vulnerable groups, namely children under 5 years of age and pregnant women, as well as the poor. The continuous existence of such huge burden due to malaria in 2010 has necessitated the implementation of large scale programs geared at eliminating malaria as a public health problem. Effective treatment of malaria requires precise laboratory diagnosis and this remains a cornerstone for global malaria control efforts. Microscopy still remains the method of choice in the diagnosis of malaria in endemic areas because it is cost effective. However correct identification of *Plasmodium* species by microscopy depends on factors, such as the experience of the microscopists, proper staining of the slides, good quality reagents, appropriate maintenance of the microscope and the time spent reading a slide. Due to these limitations, physicians are often reluctant to accept results of microscopy in such operational settings. Therefore, the reliance of diagnosis on clinical grounds alone has resulted in over diagnosis of malaria in many clinical settings in developing countries including Cameroon [3–5]. Rapid diagnostic tests have considerable potential as a tool to improve the diagnosis of malaria in endemic settings [5, 6]. Due to the rapidity of the test and availability of the results for clinical care, RDTs have been positively recommended by the World Health Organization (WHO) when reliable microscopy is not available [7].

There is increasing interest in introducing RDTs for diagnosing malaria and improving malaria case management with artemisinin-based combination therapy (ACT), as studies show significant improvements in malaria control following implementation [8]. In 2010, forty-two African countries reported deployment of 11 million ACT at community level, with only a small proportion in the community receiving parasite confirmation test at community level [2]. Clearly, the WHO underscores the importance of expanding malaria diagnosis at community level as demonstrated in Zambia with scaling up of RDT use during the period 2004–2009 [2]. The benefits of such a strategy include reduction in expenditures on anti-malarial drugs, improved patient outcomes for non-malarial fevers and curbing of drug resistance [15, 16]. The Government of Cameroon planned to introduce RDTs into communities in 50 pilot Health Districts in the national territory in 2011 as part of a Global Fund supported project for deploying ACT for case management at community level. However, there has been little or no previous report on the field performance of different RDTs recommended by the WHO and available in the Cameroon market to properly inform procurement. It was, therefore, necessary to evaluate the performance of RDTs available in the Cameroon market. This study therefore aimed to determine and compare the diagnostic accuracy of 22 different rapid diagnostic tests available in Cameroon at that time.

## Methods

### Participants

The study population was made up of male and female febrile patients aged 1 year to 16 years suspected of a malaria infection in six busy peripheral health centres in Yaoundé. A patient was considered for enrolment into the study if he/she fulfilled the following criteria: willingness to donate 5 mls of blood after informed consent. If the patient sample was positive by microscopy, consent/assent was requested to participate. If the patient’s blood sample was negative by microscopy, the patient was still requested to consent. This procedure was followed consecutively until the required number of malaria positive and negative samples was obtained. Those performing the microscopy at the health centres were trained to collect, prepare, read and quantify malaria smears by light microscopy.

### Setting

Malaria transmission in Yaoundé is year round with four climatically distinct seasons composed of 2 short and two long dry and raining seasons respectively. Peak transmission occurs in the beginning of the rainy season. This assessment was conducted in April when transmission of malaria was expected to be at its peak.

### Specimen collection

In each centre, 5 ml of whole blood was collected from consenting febrile patients by venous puncture into pre-labelled EDTA tubes. For each blood specimen, about 200 μl was used to prepare a thick film and a thin blood smear for malaria parasite density determination and speciation. Microscopy was used as the reference test. This test was chosen because it is considered as the reference method for malaria diagnosis by the WHO. Blood smears were stained with freshly prepared 3 % Giemsa solution according to standard procedures. To confirm microscopy diagnosis at the health centre, a new diagnosis was performed by two experienced and certified microscopists from the Biotechnology Centre and the Yaoundé University Teaching Hospital. They were blinded to the health centre diagnosis. The thick smears were used to detect the presence of *Plasmodium* infection. The thin smears were considered negative if no parasite was seen in 100 oil-immersion fields from two independent readings. A third reading was performed in case of positive/negative discordance for asexual stages.

### Rapid diagnostic tests

The RDTs in the study were obtained from major local distributors of clinical rapid diagnostic tests and other clinical laboratory suppliers in Cameroon. In total, twenty-two different RDTs used for malaria diagnosis and present in the market in 2010 were obtained. Selection of the RDTs was based on their presence in the Cameroon market and distributor’s willingness to participate in the assessments. The test cassettes or strips were of traceable quality (standard supplier, used within the shelf life of the product). History of proper storage and transport conditions from manufacturers could not be guaranteed.

The technical specifications of the various RDTs are presented as supplemental material (Additional file 1). Nine of the RDT kits detect only *Plasmodium falciparum,* while the rest had detection analytes for *P. falciparum* and PAN representing other *Plasmodium* species. The test devices were mostly plastic lateral flow cassettes (18) and dipsticks (4). Most of the RDTs kits contained specimen transfer/reagent transfer devices except stated in the instructions to use a specific transfer device like a micropipette. Test antigens used in the kits were *P. falciparum* specific histidine rich protein II (HRP2), lactate dehydrogenase (LDH) and aldolase in order of frequency of occurrence in RDTs.

### RDT test procedure and evaluating test results

**Fig. 1:**
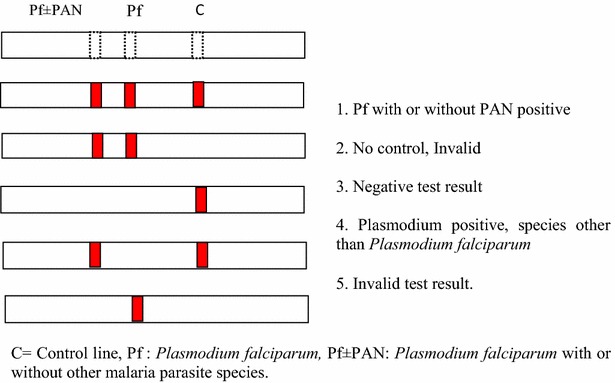
Test line configuration for HRPII based RDTs used in our assessments and their interpretation. Line configurations differed slightly depending on target antigen. In this study target antigens for RDTs included *Plasmodium* LDH, HRP2 and aldolase. Invalid test cassettes were noted and replaced during the assessments. *C*: Control line, *Pf*: *Plasmodium falciparum*, *Pf* *+* *PAN*: *Plasmodium falciparum* with or without other malaria parasite species

Evaluation of the 22 RDTs was performed and interpreted according to the manufacturers’ instructions presented in the inserted inserts. Three technicians performed the tests. These technicians were not trained, but could interpret the procedures stated in the insert. In the case where the procedure required prior preparation of reagents or the use of multiple devices, each procedure technician consistently performed the same task each time to ensure uniformity in test performance. They were blinded to each other’s results and to the results of microscopy. For each RDT the result was classified as negative, positive or invalid (Fig. 1). All tests without a control line were considered invalid by the test reader and the test repeated with a new RDT of the same code.

### Assessment of ease of use and interpretation of RDTs

The study laboratory technicians who performed the RDTs ranked the 22 tests independently in order of preference where 2 corresponded to their most preferred test and “0” to their least preferred based on a questionnaire response on a Likert scale from 0(poor) to 3(good) to 5(best): Ease and safety of taking blood; ease of adding reagents including the complexity of material/handling/transfer technique and device required and ease of interpretation of insert instructions. In addition, assessors of RDT results were asked to rank the tests independently based ease of interpretation defined by the position of bands, labeling, band intensity, background and time to interpretation of results.

### Sample size, data management and analysis

This assessment was a passive case detection survey. For malaria RDT under the study setting to perform well, the sensitivity should be at least 90 % and the specificity 80 %. expert opinion was sought that indicated a likelihood ratio of the positive test of at least 2 was clinically useful in ruling in malaria diagnosis with a good RDT. The likelihood ratio was used as our as our diagnostic test index because it does not change with pre-test probability. Therefore the 95 % confidence interval to exclude the likelihood ratio estimate using the values of the parameters provided above (Sensitivity/1-specificity) was calculated. This is given by the expression

$${\text{LR}}_{x} = \exp \left( {{\text{In}}\frac{{p_{1} }}{{p_{2} }} \pm 1.96\sqrt {\frac{{1 - p_{1} }}{{p_{1} n_{1} }} + \frac{{1 - p_{2} }}{{p_{2} n_{2} }}} } \right)$$

where *P*_1_ and *P*_2_ are sensitivity and (1-specificity) respectively and n1 = n2 = sample size. In this way the sample size was estimated at 100.

The data was checked for inconsistencies in data entry such as missing values and then used to calculate the sensitivity, specificity, the predictive values of the negative and positive RDT tests as well as the likelihood ratios and their 95 % confidence intervals. Inter-observer variations in the RDT test assessments were estimated by calculating for each operational parameter the Cohen’s kappa (κ) statistic as follows:

$$Kappa = \frac{A - B}{C - B}$$

where A = agreement observed, B = agreement by chance = 50 % of times, C = agreement possible = 100 %.

A 95 % confidence interval (95 % CI) was calculated for each κ value and the overall agreement was evaluated as the weighted κ value with its 95 % confidence interval.

### Ethics statement

This study was carried out under the auspices of the National Malaria Control Program with ethical clearance from the national ethics committee on health research in humans. Febrile patients whose left over blood specimen from routine medical exam were used provided verbal consent by phone. Information about the research was explained to each of one hundred participants/guardians by the consultant of the health centre either by phone or face to face for those who could come to the clinics. Participation was entirely voluntary.

## Results and discussion

Overall, each of 22 different RDTs were tested on a total of ninety-seven venous blood samples. Three of the RDT test types were dipsticks while the rest were strips in rectangular cassettes.

### Sensitivity, specificity and predictive values of the rapid tests

Results of the RDT evaluation are presented in Additional file 2. From this table, of all RDTs tested, (21/22) 95.4 % had sensitivity values greater than 90 % consistent with the WHO recommendations [7]. Among these RDTs with greater than 90 % sensitivity, 7/9 (77.8 %) were “Pf Only” RDTs while the rest (14/22; 63.6 %) were “Pf + PAN” RDTs. with sensitivity values greater than 90 % indicating that there seems to be more of highly performing “Pf Only” RDTs than “Pf + PAN” RDTs Furthermore, only 6/13 (46.1 %) of “Pf + PAN” RDTs had sensitivity values less than 80 %. Two “Pf + PAN” RDTs had sensitivity less than 55 %. Considering specificity, only one “Pf Only” RDT had specificity greater than 90 %. The “Pf Only” RDTs had poor specificities with values around 60 %. On the other hand, only about 8 (36.3 %) “Pf + PAN” RDTs had sensitivity values greater than 90 %. Generally, RDTs with high sensitivity had low specificities and vice versa. A few RDTs (6/23 or 23 %) had sensitivities and specificity values of at least 80 % and these were all “Pf + PAN” RDTs indicating that these RDT types seems to be better in diagnosing malaria when it is important to know if other species are contributing to the infection. Among all RDT analytes, the pLDH consistently showed higher sensitivity and specificity compared to the pHRP2. The predictive values of the positive test were generally below 80 % for 18 RDTs (78.2 %) among which only 2 were “Pf Only” RDTs. Of all RDTs tested, 11(47.8 %) had predictive values of the negative test greater than 80 %. Of these 11 RDTs, only one was a “Pf Only” RDT while among the “Pf + PAN” RDTs with greater than 80 % predictive value of the negative test, only 1(7.6 %) was represented by the pHRP2 analyte, comparing pHRP2 and pLDH/pAldolase on the “Pf + PAN” RDTs. This indicates that on the “Pf + PAN” RDTs, the PAN analyte on “Pf + PAN” RDTs seems to be better in excluding infections with *P. falciparum* compared to pfHRP2 on the same RDTs.

The majority of RDTs with good sensitivity had specificity less than 75 %. Only 6/14 or 42 % had sensitivity and specificity values greater than 80 %. Even though the sensitivity of an RDT is most often considered as the key parameter in clinical case management in malaria, it is desirable that the test analyte detects the antigens for which it is intended. One of the challenges associated with the use of RDTs is cross reactivity with antigens of a similar nature but from a different source [18]. Arguably, that the use of RDTs in case management provides added benefit of identifying patients who would otherwise have been missed if diagnosis relied on microscopy alone [19] eliminating the problem of overdiagnosis. Indeed, a recent exit poll involving malaria treated patients in Cameroon observed that over 50 % of patients were overtreated for malaria [5]. It is important that the RDTs used for widespread identification of malaria patients within integrated programmes have good specificity values to avoid a negative impact of the implemented RDT for facility and community management of fevers.

### Performance of analytes in RDTs

Tests that target HRP of *P. falciparum* demonstrated higher parasite detection rates compared to other analytes among all RDTs in this evaluation. However, tests that target LDH performed better when comparing both sensitivity and specificity for “Pf + PAN” RDTs. These findings are in agreement with the WHO based evaluation of RDTs for informed recommendations [7]. Therefore, tests that target HRP2 present in “Pf Only” RDTs appear to be better suited for the purpose of detecting the majority of malaria infections in the community. Furthermore, tests that were PAN specific had better predictive values of the positive and negative tests as well as the likelihood ratios of positive and negative tests compared to tests that detected HRP2 only. In the case where the additional objective would be to identify the presence of other species of parasites, test systems that include pLDH in association with HRP2 in this evaluation will be preferable. *Plasmodium falciparum* is the dominant species in our geo-ecological settings, but mixed parasite populations have been observed previously by our group to be circulating in some regions. In Bangolan, NW Cameroon where this observation was made, infections either consisted of *P. falciparum* coexisting with *Plasmodium malariae* (in 70 % of cases) or non-*P. falciparum* (*P. malariae* in 30 % of cases) [23]. Therefore, using a “Pf Only” RDT based on detection of HRP2 will miss 30 % of single infections with non-falciparum parasites. In addition, there is the possibility of gene deletion isolates that do not express HRP-2 [20], although it has not been examined among parasite isolates from Cameroon and the same evidence for pLDH has not yet been found.

### Rapid diagnostic test agreement

The weighted agreement between the two observers involved in reading the RDT results was quantified as the weighted Kappa value calculated for all test results irrespective of whether the test was a “Pf Only” or “Pf + PAN” RDT type. The weighted Kappa value obtained was 67.5 % (95 % CI 64.6–70.3 %), corresponding to a moderately agreement between the assessors under our field condition. However, the agreements were better for some tests over others. For example, good agreement was obtained for more “Pf + PAN” RDTs than for “Pf Only” RDTs. SD Bioline Pf + PAN test cassette had the best agreement (72.6, 95 % CI 58.9.6–86.4), based on the detection of both Pf and PAN while Clearview Malaria Combo was worst (54.9, 95 % CI 38.8–71.2).

### Operational characteristics

Based on the assessment of operational and technical characteristics of the 22 RDTs, differences in the test presentation, package contents, blood lancing and transfer devices, variations in insert instructions of use and safety disposal, procedures for test performance and results interpretation were observed. Additional file 3 provides a classification fo RDTs based on some operational characteristics. With the plethora of RDT tests commercially available today, even tests pre-qualified by the WHO and used in this assessment are very different in terms of their presentation, increasing the risk of error by the end user. Challenges ranging from the quality of information in inserts, configuration of test and control lines, missing information or components in the test kit, etc. all pose serious deficits that adversely influence the performance of RDTs at point of care and have also been reported recently [22]. Therefore, it is highly recommended that while efforts are being made to increase access of RDTs at the point of care, finding a way of providing a simple, informed and systematic presentation guideline and test to be followed by manufacturers of malaria RDTs destined for endemic countries is important.

Because of the free distribution of long-lasting insecticide-treated bed nets since 2011 to almost every household in Cameroon, a rapid decrease in the prevalence of malaria in many areas in Cameroon is expected. Low intensity infections may pose a diagnostic challenge for malaria surveillance purposes in the future due to shifting disease control priority, necessitating nucleic acid based tests.

### Study limitations

This study has several potential limitations. Firstly, the results of rapid diagnostic tests vary as a function of parasite density [18]. It would have been valuable to classify RDTs based on parasite density to better compare results with the WHO standards. In addition, the results also assumed manufacturer reported stability. The present assessments were performed during a season when the minimal temperature in Yaoundé varied between 25 and 29 °C. Further studies in which difference RDT performances will be compared in different real life environmental conditions will be very helpful to evaluate other stability parameters affecting the performance and decay of RDTs.

## Conclusion

Of all RDTs tested, 94.2 % (21/22) had sensitivity values greater than 90 % among which 14 (63.6 %) were ‘Pf + PAN’ RDTs. The specificity was generally lower than the sensitivity for all RDTs, and poorer for “Pf Only” RDTs. The predictive values and likelihood ratios were better for non-HRP2 analytes for “Pf + PAN” RDTs. Rapid diagnostic tests which incorporate histidine rich protein II and lactate dehydrogenase are appropriate for settings. There was moderate agreement that SD Bioline Malaria Antigen P.f/Pan, ACON Malaria P.f/pan and Parascreen pf + PAN and CareStart™ Malaria HRP2/pLDH Combo Test were most preferred in order of merit among “Pf + PAN” RDTs while SD Bioline Malaria Antigen P.f and ParaHIT^®^*pf* HRPII the most preferred among “Pf Only” RDTs. Among the dipsticks, “Pf + PAN” ParaHIT Total Dipstick was preferred. Harmonizing RDT test presentation and procedures would prevent mistakes of test performance and interpretation.

## Additional files

10.1186/s12936-016-1085-0 This Table represents the different RDTs present in the Cameroonian Market and used to evaluate accuracy under field conditions. Nineteen of the RDTs were of the lateral flow format while two were dipsticks. The assignment of codes for each RDT was purely random and this was done by an independent team member not involved with either performing the test or the reading of RDT test results.
10.1186/s12936-016-1085-0 This table presents the aggregate results of the accuracy of rapid tests evaluated in the study.
10.1186/s12936-016-1085-0 This table shows a classification of RDTs on the basis of some operational characteristics. Assessment of the suitability for field use included the simplicity of performing the test based on the insert instructions and illustrations on the one hand and the ease of interpretation of results within the time frame stated in the insert. The provision or requirement of additional materials like testubes, pipettes etc. were also taken into consideration in the final assessments. The scale used for classifying RDTs based on operational characteristics is alsho shown.

## Abbreviations

RDT: rapid diagnostic test
pLDH: plasmodium lactate dehydrogenase
HRP2: histidine rich protein II
WHO: World Health Organization
PPV: positive predictive value
NPV: negative predictive value
