# Supplementary material for: Field evaluation of the 22 rapid diagnostic tests for community management of malaria with artemisinin combination therapy in Cameroon
Source: Malar J. 2016 Jan 20;15:31. doi: 10.1186/s12936-016-1085-0 (PMC4721050; doi:10.1186/s12936-016-1085-0)
Supplement: Supplementary file 1 — 10.1186/s12936-016-1085-0 This Table represents the different RDTs present in the Cameroonian Market and used to evaluate accuracy under field conditions. Nineteen of the RDTs were of the lateral flow format while two were dipsticks. The assignment of codes for each RDT was purely random and this was done by an independent team member not involved with either performing the test or the reading of RDT test results. [file 12936_2016_1085_MOESM1_ESM.doc]

**Table S1: Malaria RDTs Kits Evaluated and their Corresponding Codes Assigned.**

| **Code** | **RDTs test kits** | **Antigen detected** | **Sample volume** | **Type of device** | **Incubation time** | **Limitations/advantages** | **Manufacturer** |
| --- | --- | --- | --- | --- | --- | --- | --- |
| A | ICT Malaria Test Cassette Combo | P.f HRPII and/or another pan specific (P.f., P.m., P.o. and P.v.) malaria antigen | 5μl of capillary or venous blood | Plastic cassette | 15 minutes | Cannot differentiate between mixed infections with P.f. and P.m., P.o. or P.v.  Test may remain positive after treatment | ICT Diagnostics. |
| B | SD Bioline Malaria Antigen P.f/Pan | P.f. HRPII and/or pan specific pLDH | 5μl of capillary or venous blood | Plastic cassette | 15 minutes (up to 30 minutes) | Cannot differentiate between mixed infections with P.f. and P.m., P.o. or P.v.  In P.f infections, test may remain positive after treatment | Standard diagnosis, INC. |
| C | CareStart^TM^ Malaria HRP2 | P.f. HRPII | 5μl of capillary or venous blood | Plastic cassette | 15 minutes | Detects only P.f. infections  Test may remain positive after treatment | Access Bio, Inc. |
| D | ACON Malaria P.f/pan | P.f. HRPII and/or pan specific Aldolase | 10μl of capillary or venous blood | Plastic cassette | 15 minutes | Cannot differentiate between mixed infections with P.f. and P.m., P.o. or P.v. | ACON Laboratories, Inc. |
| E | SD Bioline Malaria Antigen P.f | P.f. HRPII and/or pan specific pLDH | 5μl of capillary or venous blood | Plastic cassette | 15 minutes (up to 30 minutes) | Detects only P.f. infections  Test may remain positive after treatment | Standard diagnosis, INC. |
| F | Advanced Quality^TM^ Malaria (P.f.) Poct Test | P.f. HRPII | 10μl of capillary or venous blood | Plastic cassette | 15 minutes | Detects only P.f. infections  Test may remain positive after treatment | In Tec PRODUCTS, INC. |
| G | ICT MALARIA P.f. Test Cassette | P.f. HRPII | 5μl of capillary or venous blood | Plastic cassette | 15 minutes | Detects only P.f. infections  Test may remain positive after treatment | ICT Diagnostics |
| H | Wondfo One Step Malaria P.f/Pan Whole Blood Test | P.f. HRPII and/or pan specific pLDH | 5μl of capillary or venous blood | Plastic cassette | 15 minutes | Cannot differentiate between mixed infections with P.f. and P.m., P.o. or P.v.  In P.f. infections, test may remain positive after treatment | GUANGZHOU WONDFO BIOTECH CO., LTD |
| I | ParaHIT® *f* | P.f. HRPII | 8μl of capillary or venous blood | Plastic cassette | 15 minutes | Detects only P.f. infections  Test may remain positive after treatment  Background clearance may be delayed for 15-20 minutes in few fresh samples and in stored samples | Span Diagnostics Ltd. |
| J | FIRST RESPONSE® MALARIA pLDH/HRP2 Combo Test | P.f. HRPII and/or pan specific pLDH | 5μl of capillary or venous blood | Plastic cassette | 20 minutes | Cannot differentiate between mixed infections with P.f. and P.m., P.o. or P.v.  In P.f. infections, test may remain positive after treatment | Premier Medical Corporation Ltd |
| K | Parascreen^TM^ | P.f. HRPII and/or pan specific pLDH | 5μl of capillary or venous blood | Plastic cassette | 20 minutes | Cannot differentiate between mixed infections with P.f. and P.m., P.o. or P.v.  In P.f infections, test may remain positive after treatment | Zephyr Biomedicals |
| L | Parabank ^TM^ | Pan specific pLDH | 5μl of capillary or venous blood | Plastic cassette | 20 minutes | Useful to monitor the success of antimalarial therapy  Cannot differentiate between mixed infections with P.f. and P.m., P.o. or P.v. | Zephyr Biomedicals |
| M | FIRST RESPONSE® MALARIA Ag. P. falciparum (HRP2) Test | P.f. HRPII | 5μl of capillary or venous blood | Plastic cassette | 20 minutes | Detects only P.f. infections  Test may remain positive after treatment | Premier Medical Corporation Ltd |
| N | CareStart^TM^ Malaria HRP2/pLDH Combo Test | P.f. HRPII and/or pan specific pLDH | 5μl of capillary or venous blood | Plastic cassette | 20 minutes | Cannot differentiate between mixed infections with P.f. and P.m., P.o. or P.v.  In P.f infections, test may remain positive after treatment | Access Bio, Inc. |
| O | ParaHIT Total | P.f. HRPII and/or pan specific aldolase/pLDH | 8μl of capillary or venous blood | Plastic cassette | 15 minutes | Cannot differentiate between mixed infections with P.f. and P.m., P.o. or P.v.  In P.f infections, test may remain positive after treatment  Background clearance may be delayed for 15-20 minutes in few fresh samples and in stored samples | Span Diagnostics Ltd. |
| P | Paracheck® Pf | P.f. HRPII | 5μl of capillary or venous blood | Plastic cassette | 20 minutes | Detects only P.f. infections  Test may remain positive after treatment | Orchid Biomedical Systems |
| Q | CareStart^TM^ Malaria pLDH | pLDH | 5μl of capillary or venous blood | Plastic cassette | 20 minutes | Cannot differentiate between mixed infections with P.f. and P.m., P.o. or P.v.  Useful to monitor the success of antimalarial therapy | Access Bio, Inc. |
| R | Wondfo One Step Malaria P.f Whole Blood Test | P.f. HRPII | 5μl of capillary or venous blood | Plastic cassette | 15 minutes | Detects only P.f. infections  Test may remain positive after treatment | GUANGZHOU WONDFO BIOTECH CO., LTD |
| S |  |  |  |  |  |  |  |
| T | ParaHIT® *f* | P.f. HRPII | 8μl of capillary or venous blood | Dipstick | 15 minutes | Detects only P.f. infections  Test may remain positive after treatment  Background clearance may be delayed for 15-20 minutes in few fresh samples and in stored samples |  |
| U | ParaHIT Total | P.f. HRPII and/or pan specific aldolase/pLDH | 8μl of capillary or venous blood | Dipstick | 15 minutes | Cannot differentiate between mixed infections with P.f. and P.m., P.o. or P.v.  In P.f infections, test may remain positive after treatment  Background clearance may be delayed for 15-20 minutes in few fresh samples and in stored samples | Span Diagnostics Ltd. |
| V | IMMUNOQUICK MALARIA |  | 20μl of capillary or venous blood | Dipstick | Between 10-15 minutes |  | BIOSYNEX |
