# Supplementary material for: Field evaluation of the 22 rapid diagnostic tests for community management of malaria with artemisinin combination therapy in Cameroon
Source: Malar J. 2016 Jan 20;15:31. doi: 10.1186/s12936-016-1085-0 (PMC4721050; doi:10.1186/s12936-016-1085-0)
Supplement: Supplementary file 2 — 10.1186/s12936-016-1085-0 This table presents the aggregate results of the accuracy of rapid tests evaluated in the study. [file 12936_2016_1085_MOESM2_ESM.docx]

**Table S2: Accuracy evaluation of 22 rapid diagnostic tests present in the Cameroon market**

| **RDT Test Type** | **FN** | **FP** | **TN** | **TP** | **SE** | **95%CI** | **SP** | **95%CI** | **LR+** | **95%CI** | **LR-** | **95%CI** | **DOR** | **95%CI** | **PV+** | **PV-** |
| --- | --- | --- | --- | --- | --- | --- | --- | --- | --- | --- | --- | --- | --- | --- | --- | --- |
| ICT Malaria Test Cassette Combo | 3 | 17 | 35 | 42 | 0,93 | 0.82 - 0.97 | 0,67 | 0.54 - 0.78 | 2,86 | 1.92 - 4.25 | 0,10 | 0.03 - 0.30 | 28,80 | 7.8 - 106 | 0,71 | 0,33 |
| ICT PAN | 1 | 12 | 40 | 44 | 0,98 | 0.88 - 1.0 | 0,77 | 0.64 - 0.86 | 4,24 | 2.58 - 7.0 | 0,02 | 0.004 - 0.22 | 146,00 | 18 - 1179 | 0,79 | 0,23 |
| SD Bioline Malaria Antigen P.f/Pan | 1 | 18 | 34 | 44 | 0,98 | 0.88 - 1.0 | 0,65 | 0.52 - 0.77 | 2,83 | 1.94 - 4.12 | 0,03 | 0.005 - 0.24 | 83,00 | 10.56 - 653 | 0,71 | 0,35 |
| SD Bioline PAN | 5 | 4 | 48 | 40 | 0,89 | 0.77 - 0.95 | 0,92 | 0.81 - 0.97 | 11,60 | 4.5 - 29.8 | 0,12 | 0.05 - 0.78 | 96,00 | 24.1 - 381 | 0,91 | 0,08 |
| CareStart^TM^ Malaria HRP2 | 2 | 17 | 36 | 42 | 0,95 | 0.85 - 0.99 | 0,68 | 0.55 - 0.79 | 3,00 | 2.0 - 4.4 | 0,07 | 0.02 - 0.26 | 44,50 | 9.6 - 205.6 | 0,71 | 0,32 |
| ACON Malaria P.f/pan | 2 | 17 | 35 | 43 | 0,96 | 0.85 - 0.99 | 0,67 | 0.54 - 0.78 | 2,90 | 2.0 - 4.3 | 0,07 | 0.02 - 0.26 | 44,30 | 9.6 - 204.7 | 0,72 | 0,33 |
| ACON PAN | 1 | 20 | 51 | 25 | 0,96 | 0.81 - 0.99 | 0,72 | 0.6 - 0.81 | 3,40 | 2.3 - 4.99 | 0,05 | 0.01 - 0.37 | 63,80 | 8.1 - 502.5 | 0,56 | 0,28 |
| SD Bioline Malaria Antigen P.f | 1 | 19 | 33 | 44 | 0,98 | 0.88 - 1.0 | 0,63 | 0.50 - 0.75 | 2,68 | 1.87 - 3.84 | 0,04 | 0.01 - 0.25 | 76,40 | 9.7 - 600.1 | 0,70 | 0,37 |
| Advanced Quality^TM^ Malaria (P.f.) Poct Test | 14 | 27 | 25 | 31 | 0,69 | 0.54 - 0.80 | 0,48 | 0.35 - 0.61 | 1,33 | 0.96 - 1.84 | 0,65 | 0.39 - 1.09 | 2,05 | 0.89 - 4.7 | 0,53 | 0,52 |
| Advanced Quality^TM^ PAN | 17 | 18 | 33 | 29 | 0,63 | 0.49 - 0.75 | 0,65 | 0.51 - 0.76 | 1,79 | 1.16 - 2.75 | 0,57 | 0.37 - 0.81 | 3,13 | 1.36 - 7.17 | 0,62 | 0,35 |
| ICT MALARIA P.f. Test Cassette | 6 | 17 | 35 | 39 | 0,87 | 0.74 - 0.93 | 0,67 | 0.54 - 0.78 | 2,65 | 1.77 - 3.96 | 0,20 | 0.09 - 0.43 | 13,38 | 4.75 - 37.73 | 0,70 | 0,33 |
| Wondfo One Step Malaria P.f/Pan Whole Blood Test | 10 | 5 | 47 | 35 | 0,78 | 0.64 - 0.87 | 0,90 | 0.79 - 0.96 | 80,90 | 3.47 - 18.88 | 0,25 | 0.14 - 0.43 | 32,90 | 10.32 - 104.9 | 0,88 | 0,10 |
| Wondfo One Step Malaria PAN | 12 | 7 | 45 | 33 | 0,73 | 0.59 - 0.84 | 0,87 | 0.74 - 0.93 | 5,44 | 2.68 - 11.10 | 0,31 | 0.19 - 0.51 | 17,68 | 6.28 - 49.8 | 0,83 | 0,13 |
| FIRST RESPONSE^®^ MALARIA pLDH/HRP2 Combo Test | 5 | 13 | 39 | 40 | 0,89 | 0.77 - 0.95 | 0,75 | 0.62 - 0.85 | 3,56 | 2.20 - 5.76 | 0,15 | 0.06 - 0.34 | 24,00 | 7.82 - 73.7 | 0,75 | 0,25 |
| FIRST RESPONSE PAN | 2 | 15 | 37 | 43 | 0,96 | 0.85 - 0.99 | 0,71 | 0.58 - 0.82 | 3,30 | 2.15 - 5.10 | 0,06 | 0.02 - 0.25 | 53,00 | 11.38 - 247.3 | 0,74 | 0,29 |
| Parascreen^TM^ | 2 | 15 | 37 | 43 | 0,96 | 0.85 - 0.99 | 0,71 | 0.74 - 0.93 | 5,40 | 2.68 - 11.10 | 0,31 | 0.19 - 0.51 | 17,68 | 6.28 - 49.8 | 0,74 | 0,29 |
| Parascreen^TM^ PAN | 2 | 7 | 45 | 43 | 0,96 | 0.85 - 0.98 | 0,87 | 0.74 - 0.93 | 7,10 | 3.55 - 14.18 | 0,05 | 0.013 - 0.2 | 138,20 | 27.2 - 702 | 0,86 | 0,13 |
| CareStart^TM^ Malaria HRP2/pLDH Combo Test | 1 | 18 | 34 | 44 | 0,98 | 0.88 - 1.0 | 0,65 | 0.52 - 0.77 | 2,83 | 1.94 - 4.12 | 0,03 | 0.005 - 0.24 | 83,10 | 10.56 - 653.9 | 0,71 | 0,35 |
| Carestart^TM^ PAN | 8 | 2 | 50 | 37 | 0,82 | 0.69 - 0.91 | 0,96 | 0.87 - 0.99 | 21,38 | 5.45 - 83.77 | 0,19 | 0.10 - 0.35 | 115,00 | 23.2 - 576.6 | 0,95 | 0,04 |
| Parabank ^TM^ | 4 | 4 | 48 | 41 | 0,91 | 0.79 - 0.96 | 0,92 | 0.82 - 0.97 | 11,84 | 4.60 - 30.5 | 0,10 | 0.04 - 0.25 | 123,00 | 28.9 - 522 | 0,91 | 0,08 |
| FIRST RESPONSE^®^ MALARIA Ag. P. falciparum (HRP2) Test | 1 | 19 | 33 | 44 | 0,98 | 0.88 - 1.0 | 0,63 | 0.50 - 0.75 | 2,68 | 1.87 - 3.84 | 0,04 | 0.005 - 0.25 | 76,40 | 9.73 - 600 | 0,70 | 0,37 |
| ParaHIT Total | 1 | 21 | 31 | 44 | 0,98 | 0.88 - 1.0 | 0,60 | 0.46 - 0.72 | 2,42 | 1.74 - 3.38 | 0,04 | 0.005 - 0.26 | 65,00 | 8.3 - 508.6 | 0,68 | 0,40 |
| ParaHIT Total PAN | 2 | 12 | 40 | 43 | 0,96 | 0.85 - 0.98 | 0,77 | 0.63 - 0.86 | 4,14 | 2.51 - 6.83 | 0,06 | 0.15 - 0.226 | 71,70 | 15.1 - 340.2 | 0,78 | 0,23 |
| Wondfo One Step Malaria P.f Whole Blood Test | 1 | 13 | 39 | 44 | 0,98 | 0.88 - 1.0 | 0,75 | 0.62 - 0.84 | 3,91 | 2.4 - 6.28 | 0,03 | 0.004 - 0.20 | 132,00 | 16.5 - 1055 | 0,77 | 0,25 |
| Wondfo One Step PAN | 5 | 4 | 46 | 40 | 0,89 | 0.32 - 0.72 | 0,92 | 0.95 - 1.00 | N/A | N/A | 0,48 | 0.304 - 0.75 |  |  | 0,91 | 0,08 |
| Paracheck^®^ Pf | 1 | 19 | 33 | 44 | 0,98 | 0.88 - 1.0 | 0,63 | 0.50 - 0.75 | 2,68 | 1.87 - 3.84 | 0,04 | 0.005 - 0.25 | 76,40 | 9.7 - 600.1 | 0,70 | 0,37 |
| CareStart pLDH | 1 | 11 | 40 | 45 | 0,98 | 0.89 - 1.00 | 0,78 | 0.65 to0.87 | 4,50 | 2.68 - 7.67 | 0,03 | 0.004 - 0.19 | 163,60 | 20.2 - 1324 | 0,80 | 0,22 |
| ParaHit Total* | 6 | 15 | 37 | 39 | 0,87 | 0.74 - 0.94 | 0,71 | 0.58 - 0.82 | 3,00 | 1.93 - 4.67 | 0,19 | 0.09 - 0.40 | 16,33 | 5.62 - 45.0 | 0,72 | 0,29 |
| ParaHit Total PAN | 8 | 10 | 42 | 37 | 0,82 | 0.69 - 0.91 | 0,81 | 0.68 - 0.89 | 4,28 | 2.41 - 7.59 | 0,22 | 0.12 - 0.41 | 19,40 | 6.94 - 54.4 | 0,79 | 0,19 |
| Clearview^®^ Malaria Combo | 3 | 13 | 39 | 42 | 0,93 | 0.82 - 0.98 | 0,75 | 0.62 - 0.85 | 3,73 | 2.3 - 6.02 | 0,09 | 0.03 - 0.27 | 42,00 | 11.12 - 158.6 | 0,76 | 0,25 |
| Clearview^®^ PAN | 24 | 1 | 51 | 21 | 0,47 | 0.33 - 0.61 | 0,98 | 0.90 - 1.0 | 24,30 | 3.4 - 173 | 0,54 | 0.41 - 0.72 | 44,60 | 5.67 - 351 | 0,95 | 0,02 |
| ParaHIT^®^ *f* | 4 | 13 | 38 | 42 | 0,91 | 0.80 - 0.97 | 0,75 | 0.61 - 0.84 | 3,58 | 2.22 - 5.78 | 0,12 | 0.05 - 0.31 | 30,70 | 9.2 - 102.3 | 0,76 | 0,25 |
| ParaHIT Total Dipstick | 1 | 16 | 36 | 44 | 0,98 | 0.88 - 1.0 | 0,69 | 0.56 - 0.80 | 3,18 | 2.11 - 14.78 | 0,03 | 0.005 - 0.22 | 99,00 | 12.52 - 782.7 | 0,73 | 0,31 |
| ParaHIT Total Dipstick PAN | 9 | 4 | 49 | 35 | 0,80 | 0.66 - 0.89 | 0,92 | 0.82 - 0.97 | 10,54 | 4.06 - 27.4 | 0,22 | 0.12 - 0.40 | 47,60 | 13.6 - 167.1 | 0,90 | 0,08 |
| IMMUNOQUICK MALARIA | 2 | 16 | 36 | 43 | 0,96 | 0.85 - 0.98 | 0,69 | 0.55 - 0.80 | 3,10 | 2.06 - 4.69 | 0,06 | 0.02 - 0.25 | 48,40 | 10.42 - 224 | 0,73 | 0,31 |

**Legend**: PAN:*Test line that detects Aldolase or Lactate dehydrogenase as target analyte secreted by all infecting malaria parasite species.**: *This rapid test was obtained from a different distributor, although manufactured by the same parent company. TN: True negative, FN: False negative, FP: False positive, TP: True positive, DOR: diagnostic odds ratio, LR+: likelihood ratio of positive test, LR-: likelihood ratio of negative test, PV+: predictive value of positive test, PV-: predictive value of negative test, SE: Sensitivity, SP: Specificity, 95%CI: 95% Confidence interval.*
