# Supplementary material for: Field evaluation of the 22 rapid diagnostic tests for community management of malaria with artemisinin combination therapy in Cameroon
Source: Malar J. 2016 Jan 20;15:31. doi: 10.1186/s12936-016-1085-0 (PMC4721050; doi:10.1186/s12936-016-1085-0)
Supplement: Supplementary file 3 — 10.1186/s12936-016-1085-0 This table shows a classification of RDTs on the basis of some operational characteristics. Assessment of the suitability for field use included the simplicity of performing the test based on the insert instructions and illustrations on the one hand and the ease of interpretation of results within the time frame stated in the insert. The provision or requirement of additional materials like testubes, pipettes etc. were also taken into consideration in the final assessments. The scale used for classifying RDTs based on operational characteristics is alsho shown. [file 12936_2016_1085_MOESM3_ESM.docx]

**Table S3: Classification of RDTs as a function of some Operational Characteristics**

| RDT | Pf only or Pf+PAN | Dipstick or Cassette | Test preference | | | | Classification of Pf+PAN RDTs | Classification of Pf Only RDTs |
| --- | --- | --- | --- | --- | --- | --- | --- | --- |
| A | Pf+PAN | Cassette | 2 | 1 | 2 | 1 | SD Bioline Malaria Antigen P.f/Pan , and and | SD Bioline Malaria Antigen P.f |
| B | Pf+PAN | Cassette | 2 | 2 | 2 | 2 | ParaHIT Total Dipstick | ParaHIT^®^ *pf* |
| C | Pf Only | Cassette | 2 | 2 | 2 | 2 | ACON Malaria P.f/pan | CareStart^TM^ Malaria HRP2 |
| D | Pf+PAN | Cassette | 2 | 2 | 2 | 2 | Parascreen pf+PAN | Parabank ^TM^ |
| E | Pf Only | Cassette | 2 | 2 | 2 | 2 | CareStart^TM^ Malaria HRP2/pLDH Combo Test | FIRST RESPONSE^®^ MALARIA Ag. P. falciparum (HRP2) Test |
| F | Pf+PAN | Cassette | 2 | 1 | 1 | 1 | Wondfo One Step Malaria P.f/Pan | Paracheck^®^ Pf |
| G | Pf Only | Cassette | 2 | 2 | 1 | 2 | FIRST RESPONSE^®^ MALARIA pLDH/HRP2 Combo Test | CareStart^TM^ Malaria pLDH |
| H | Pf+PAN | Cassette | 2 | 2 | 2 | 2 | ParaHIT Total | T |
| I | Pf Only | Cassette | 2 | 2 | 2 | 2 | Clearview^®^ Malaria Combo | ICT MALARIA P.f. Test Cassette |
| J | Pf+PAN | Cassette | 2 | 1 | 2 | 2 | Advanced Quality^TM^ Malaria (P.f.) Poct Test |  |
| K | Pf+PAN | Cassette | 2 | 2 | 2 | 2 | ICT Malaria Test Cassette Combo |  |
| L | Pf Only | Cassette | 2 | 2 | 2 | 2 | IMMUNOQUICK MALARIA |  |
| M | Pf Only | Cassette | 2 | 2 | 2 | 2 |  |  |
| N | Pf+PAN | Cassette | 2 | 1 | 2 | 1 |  |  |
| O | Pf+PAN | Cassette | 2 | 0 | 1 | 1 |  |  |
| P | Pf Only | Cassette | 2 | 1 | 2 | 1 |  |  |
| Q | Pf Only | Cassette | 2 | 2 | 1 | 1 |  |  |
| R | Pf+PAN | Cassette | 2 | 2 | 1 | 1 |  |  |
| S | Pf+PAN | Dipstick | 1 | 1 | 1 | 1 |  |  |
| T | Pf Only | Dipstick | 1 | 2 | 1 | 2 |  |  |
| U | Pf+PAN | Dipstick | 1 | 2 | 2 | 2 |  |  |
| V | Pf Only | Dipstick | 1 | 1 | 2 | 2 |  |  |

Below the criteria for classifying RDTs based on operational characteristics is presented.

|  | 1 | 2 | 3 | 4 | 5 | comments |
| --- | --- | --- | --- | --- | --- | --- |
| **Ease of use** |  |  |  |  |  |  |
| Ease and safety of taking blood | Very poor | poor | good | Fairly good | best |  |
| ease of adding reagents including the complexity of material/handling/transfer device | Very poor | poor | good | Fairly good | best |  |
| **Ease of interpretation** | 1 | 2 | 3 | 4 | 5 |  |
| Background clearance | Very poor | poor | good | Fairly good | best |  |
| Band intensity |  |  |  |  |  |  |
| Cassette presentation |  |  |  |  |  |  |
| Test line position |  |  |  |  |  |  |
| Line labelling |  |  |  |  |  |  |
| **Test preference** | Least preferred | preferred | Most preferred |  |  |  |
|  |  |  |  |  |  |  |

**Legend:** Test preference was based on aggregate score for different categories of both ease of use and ease of interpretation. A score of 0-5 was considered least preferred, 5-10 preferred and 11-15 most preferred.
